# Exploring the relationship between *Plasmodium falciparum* genetic diversity and antimalarial drugs resistance markers in a malaria-endemic region of Burkina Faso

**DOI:** 10.11604/pamj.2024.48.118.43505

**Published:** 2024-07-18

**Authors:** Moustapha Nikiema, Issiaka Soulama, Charles Quaye, Hamidou Ilboudo, Seni Nikiema, Justine Kabore, Clarisse Dah, Ali Sie, Athanase Badolo, Awa Gneme

**Affiliations:** 1 Centre de Recherche en Santé de Nouna (CRSN), Nouna, Burkina Faso,; 2 Université Joseph KI-ZERBO (UJKZ), Ouagadougou, Burkina Faso,; 3 Institut de Recherche en Sciences de la Santé (IRSS), Bobo-Dioulasso, Burkina Faso,; 4 Noguchi Memorial Institute for Medical Research (NMIMR), Accra, Ghana,; 5 Unité de Recherche Clinique de Nanoro (URCN), Nanoro, Burkina Faso

**Keywords:** *Plasmodium falciparum*, genetic diversity, antimalarial, resistance markers, Burkina Faso

## Abstract

**Introduction:**

the diversity of Plasmodium falciparum genotypes affects the dynamics of malaria transmission and is thought to be one of the factors hampering malaria control efforts. This study aimed to investigate the relationship between Plasmodium falciparum genetic diversity and chloroquine and sulfadoxine-pyrimethamine resistance markers in malaria endemic areas of Burkina Faso.

**Methods:**

in a cross-sectional study, populations residing in Nouna health district were randomly recruited. Blood samples were used for microscopic malaria diagnosis, and genetic polymorphism alleles of msp1 and msp2 genotyping by nested PCR. Restricted fragment length polymorphism analysis was used to identify antimalarial resistance markers. Logistic regression analysis explored the association between msp1/msp2 alleles and antimalarial drug resistance markers. ANOVA was used to explore the association between the mean complexity of infection (mCOI) and prevalence of resistance markers.

**Results:**

the overall prevalence of Plasmodium falciparum infection was 27.1%. The proportions of K1, MAD20, RO33, FC27, 3D7 individuals with mutations in the pfcrt76T gene were 4.3%, 6.9%, 7.0%, 6.8% and 7.1% respectively. Those with mutations in pfmdr1 were 2.7%, 2%, 2.3%, 6.8% and 7.1%. No significant associations were detected between msp1/msp2 alleles and chloroquine or sulfadoxine-pyrimethamine resistance markers. However, the mean complexity of infection (mCOI) was significantly higher in individuals with the pfcrt76T mutation.

**Conclusion:**

overall, this study showed that the genetic diversity of Plasmodium falciparum does not significantly affect the presence of antimalarial drug resistance genes. The competition between different strains (polyclonality) of the parasite within the host was probably unfavorable for mutant strains.

## Introduction

In highly malaria-endemic settings, asymptomatic and symptomatic patients harbor diverse clones of *Plasmodium falciparum*, that have been reported to be beneficial for enhancing intraspecific immunity [1]. However, this high genetic diversity is often detrimental to malaria control, leading to intense intra-host competition, increased gametocyte production, and the emergence of virulent parasite strains that are resistant to antimalarial drugs [2]. Indeed, the severity of *Plasmodium falciparum* infection and treatment failure are partly explained by its genetic polymorphisms [3]. Sub-Saharan countries recorded 249 million malaria cases with 608,000 deaths in 2022 [4]. Approximately 10% of treatment failures due to mutations are reported annually in endemic areas [5]. Among the genes involved in polymorphisms, the merozoite surface proteins 1 and 2 are the most widely studied for the understanding of treatment failure due to new infections and also for the molecular monitoring of antimalarial resistance [6-8]. The intensity of malaria transmission in non-endemic areas can be assessed using malaria prevalence, which is the number of parasites in one microliter of infected blood [9]. However, this approach does not apply to high malaria transmission settings. Other approaches, such as the use of a multiplicity of infection and entomological inoculation rates, can be used to easily evaluate transmission under any condition [10]. In Burkina Faso, several interventions strategies are being implemented to target the vectors and the parasite to reduce the number of cases and deaths and control the disease [11]. These methods include the distribution and use of long-lasting insecticidal nets (LLINs), indoor residual spraying (IRS), seasonal chemoprevention in children under five years of age, intermittent presumptive therapy (IPT) in pregnant women and the availability of rapid diagnostic tests (RDT) in peripheral health facilities [12,13]. Obviously, these various interventions, by reducing malaria morbidity and mortality, can affect the genetic diversity of the parasite and even the dynamics of resistant parasites [14]. Alongside these interventions, the emergence and spread of resistance to antimalarial drugs such as chloroquine, sulfadoxine, pyrimethamine and, more recently, artemisinin derivatives (in Southeast Asia and in East Africa), increase the risk of compromising the efficacy, seems to be a reality today, particularly for ACTs now recommended by the WHO for uncomplicated malaria treatment [15]. In general, resistance is mediated by specific genes that undergo mutations that may be natural or acquired due to drug pressure [16]. Resistance to chloroquine and amodiaquine is therefore associated with mutations in the pfcrt and *pfmdr1* genes [17]. The substitution of lysine for threonine at position 76 of the pfcrt gene is associated with chloroquine resistance, as a result of the reduced concentration of chloroquine in the food vacuole of the resistant parasite [18]. Mutation of the mammalian multidrug gene, *pfmdr1*, at position 86, from asparagine to tyrosine, increased the concentration of chloroquine inhibition (IC50) in the pfcrtK76T mutant parasite [19]. The *pfmdr1* gene is also of interest for early detection of resistance to artemisinin derivatives in countries that have adopted ACT as first-line treatment [20]. Resistance to sulfadoxine and pyrimethamine is associated with the inhibition of the action of two enzymes, including DHPS and *DHFR*, in the folate synthesis pathway [21,22]. Mutations in one of the positions 50, 51, 59, 108, 164 in the *dhfr* gene are associated with pyrimethamine resistance, while those in one of the five positions (436, 437, 540, 581, 613) in the *dhps* gene have been shown to be associated with sulfadoxine resistance [23]. However, DHFR triple mutant (51, 59, 108) and DHPS single or double mutant (437, 540) are strongly associated with sulfadoxine-pyrimethamine therapeutic failure [24]. Several molecular biology techniques are used to monitor markers of antimalarial drug resistance [25].

The relationship between genetic polymorphisms and antimalarial resistance markers is still a topic of debate [26,27]. Previous studies have noted that resistance spreads more rapidly in both high and low malaria transmission areas, while its spread is slower in intermediate regions [28]. Factors such as drug resistance mediated by a single gene and high intra-host competition can contribute to the acceleration of resistance spread [29]. Investigating the connection between genetic diversity and antimalarial drug resistance within the context of complex malaria interventions is vital for comprehending the impact of these interventions on malaria control and elimination in endemic regions. A prior study in Burkina Faso focused solely on the *pfcrt76* and *pfmdr1* genes associated with chloroquine resistance in a rural area [30]. The present study aimed to investigate the relationship between *Plasmodium falciparum* genetic diversity and genes associated with chloroquine, amodiaquine, and sulfadoxine-pyrimethamine resistance in endemic settings of Burkina Faso. Specifically, we determined the proportions of *msp1*(K1, MAD20, RO33) and *msp2* (3D7, FC27) individuals with mutations in the *pfcrt76, pfmdr1, dhfr* and *dhps* genes. We also determined the prevalence of antimalarial drug resistance markers in relation with monoclonal and polyclonal infections.

## Methods

**Study design:** a cross-sectional study was conducted in Nouna Health District to investigate the relationship between *Plasmodium falciparum* genetic diversity and antimalarial resistance genes.

**Study areas and population:** the Nouna Health District (DSN) covers the entire province of Kossi in northwestern Burkina Faso. The majority of the population of Kossi Province is subsistence farmers and pastoralists. The climate is Soudano-Sahelian, with a dry season from November to May and a rainy season from June to October. The province is located in the Sahel-Sudan zone, where malaria is transmitted seasonally over a long period (4 to 6 months) [29]. In 2020, the average temperature during the period (August to December) was 25.42°C, and the average humidity recorded was 56.30%. In the same year, the overall prevalence of malaria in the district was 28.7%. The study population consisted of individuals of both sexes, aged 3 months to 80 years, residing in the Nouna health district and randomly selected from the Demography and Health System (DHS) database of the Centre de Recherche en Santé de Nouna (CRSN).

**Variables:** the presence or absence of different *msp1*/*msp2* alleles were the categorial variables. These alleles included K1, MAD20, RO33 for *msp1* and 3D7, FC27 for *msp2*. The variables of interest included the observation of mutations in codons 76 of pfcrt, 86 of *pfmdr1*, 51, 59 and 108 of *dhfr* and 437 of *dhps* genes.

**Data source:** social demographic and health information, including sex, age, body temperature, occupation and health history were collected with informed consent. A data collection application (SurveyCTO) was used for field data collection. Capillary blood samples were collected for thick and thin smears and spotted on Whatman filter papers. Slides and dried blood spots were carefully packaged and transported to the CRSN laboratory for storage at ambient temperature for slides and at -80°C for dried blood spots. Symptomatic individuals (Body temperature =38°C) screened positive for malaria RDT were promptly treated for malaria.

**Sample size:** the formula for qualitative variables was used to estimate the sample size with an absolute error of 5% [30]. Considering the overall prevalence of malaria in Kossi province which was 28,7%, we estimated the sample size to be approximately 1000 individuals [31].

**Laboratory processing:** malaria diagnosis consisted of microscopic identification of Plasmodium species. Subsequently, parasite DNA was extracted using the QIAGEN kit according to the manufacturer's instructions from blood spots on filter paper from *Plasmodium falciparum*-positive samples. To assess genetic diversity, the extracted DNA was used for genotyping block 2 of *msp1* and block 3 of *msp2*. A previously described nested PCR protocol was used [32]. The analysis of antimalarial drug resistance markers was performed by Restricted Fragment Length Polymorphism (RFLP) using enzymatic digestion as described previously [23,30,31]. The enzymes APOI, AfIII, TSP5091, Xmnl, AvaII and FokI were used to digest the *pfcrt76*T, *pfmdr1*-86, *dhfr51, dhfr59, dhfr108* and *dhps* 437 genes overnight. The digested products were run for 45 minutes on a 2.5% agarose gel and visualized under a UV transilluminator.

**Statistical analysis:** all data were analyzed using R (version 4.2.0). The descriptive analysis consisted of calculating proportions, prevalences and frequencies. Bivari00ate analysis was used to compare the proportions of K1, MAD20 and RO33 of *msp1* and FC27 and 3D7 of *msp2* individuals with mutations in antimalarial resistance genes using the chi-squared test. The association between polymorphic alleles and antimalarial resistance genes was assessed by logistic regression analysis. ANOVA was used to assess the mean complexity of infection (mCOI) based on wild-type and mutant resistance genes. The prevalence of antimalarial genes associated with chloroquine (*pfcrt76, pfmdr1*) and sulfadoxine-pyrimethamine (*dhfr* 51,59,108 and *dhps* 437) resistance was determined. A P value of less than 5% indicated a statistically significant difference.

The complexity of infection (COI) or multiplicity of infection (MOI), defined as the number of different *Plasmodium falciparum* clones in a given infection, was determined by calculating the number of different alleles at any one locus detected in the sample [34]. The mean COI represents the average number of COIs for *msp1* and *msp2*. Multiple infections were identified when isolates exhibited more than one allelic family, whereas the presence of a single allelic family was categorized as a mono-infection. Samples with only one genotype per allelic family were considered monoclonal, whereas those with multiple genotypes per family were considered multiclonal [35].

**Ethical aspects:** the present study was approved by the Nouna Institutional Ethical Committee (N° 2021-001-/MS/SG/INSP/CRSN/CIE). The study protocol was designed and conducted by international ethical guidelines [36]. Written informed consent was obtained from the adult participants. For minor participants, written informed assent was obtained from parents or legal guardians. A codification system was used to protect and keep participants anonymous.

## Results

**Population structure:** a total of 1,049 participants were enrolled, of whom 661 (63%) were females and 388 (37%) males. The male/female sex ratio was 0.58 and the mean age was 19.1 years. Children under the age of five represented 25.3% of the participants and pregnant women represented about 2%.

**Distribution of *msp1* and *msp2* alleles and complexity of infection (COI):** of 285 samples microscopically confirmed as *Plasmodium falciparum* positive, only 4 (1.4%), 1 (0.5%) and 4 (1.4%) were found to carry single allele of K1, MAD20 and RO33, respectively. The majority of samples, 270 (96.7%), showed mixed alleles, including 20 (7.2%) with K1-MAD20, 34 (12.2%) with K1-RO33, 21 (7.5%) with MAD20-RO33 and 195 (69.8%) with K1-MAD20-RO33 from the *msp1* gene. For the *msp2* gene, 53 (19%) tested positive for 3D7, 51 (18.3%) for FC27 and 175 (62.7%) for a mixture of 3D7-FC27 alleles. Complexity of infection was significantly higher for the *msp1* genotype at 3.4, while *msp2* had a COI of 1.6. The mean complexity of infection (mCOI) was calculated to be 2.3 (95% CI 2.2-2.4).

**Prevalence of chloroquine and sulfadoxine-pyrimethamine resistance genes:** the prevalences of the *pfcrt76*T and *pfmdr1*-86Y mutant genes, which confer resistance to chloroquine and amodiaquine, were 4.2% and 7.3%, respectively. The frequencies of the *dhfr*51I, *dhfr*59R, *dhfr*108A and *dhps*437G mutations, which are associated with resistance to sulfadoxine-pyrimethamine, were 15.4%, 57.2%, 24.9% and 22.8%, respectively. No mutations were detected in codon 540E of the *dhps* gene.

**Relationship between *msp1* and *msp2* alleles and the expression of chloroquine resistance genes:** the proportions of K1 individuals with mutations in the *pfcrt76*T and *pfmdr1*-86Y genes were 4.3% and 2.7%, respectively. In individuals with the MAD20 allele, the prevalence of *pfcrt76*T and *pfmdr1*-86Y were 6.9% and 2.0%, respectively. Only 7.0% and 2.3% of individuals with the RO33 allele developed resistance in the *pfcrt76* and *pfmdr1*-86 genes, respectively. Regarding *msp2* alleles, the prevalence of *pfcrt76*T mutants was 7.1% for FC27 and 6.8% for 3D7. Multivariate analysis by logistic regression showed no association between K1 [OR = 0.94; CI (0.77-1.14); p = 0.480], MAD20 [OR = 1.05; CI (0.89-1.23); p = 0.594], RO33 [OR = 1. 06; CI (0.94 - 1.94); p=0.450], the 3D7 [0R=1.08; CI (0.81 - 1.40), p=0.600], FC27 [OR=1.05; CI (0.80 - 1.41); p=0.657] and *pfcrt76*T mutant gene (Table 1). For *pfmdr1*-86 mutant gene, no association was found with K1 [OR=1.12 CI (1.07-1.17), p=0.993], MAD20 [OR=0.83 CI (0.52-1.35), p=0.310], RO33 [OR=1.06; CI (0.94-1.94); p=0.744], 3D7 [OR=0.83; CI (0.43-1.58); p=0.520] and FC27 [OR=1.03; CI (0.64-1.66); p=0.894] (Table 2).

**Table 1:** relationship between mutant/wild type *pfcrt76* genes and K1, MAD20, RO33, FC27 and 3D7 distribution assessing by logistic regression analysis

Genes	Alleles	N°pfcrt76_mutant	N°pfcrt76_wild	OR (C.I 95 %)	P-value
**MSP1**	K1				
	Yes	16	238	0.94 (0.77-1.14)	0.480
	No	3	28	-	-
	MAD20				
	Yes	17	226	1.05 (0.89-1.23)	0.594
	No	2	40	-	-
	RO33				
	Yes	18	237	1.06 (0.94-1.94)	0.450
	No	1	29	-	-
**MSP2**	FC27				
	Yes	14	181	1.08 (0.81-1.4)	0.600
	No	5	85	-	-
	3D7				
	Yes	14	183	1.07 (0.80-1.41)	0.657
	No	5	83	-	-

**Table 2:** relationship between mutant/wild type *pfmdr1*-86 genes and K1, MAD20, RO33, FC27 and 3D7 distribution analyzed by logistic regression

Genes	Alleles	N°pfmdr1-86_mutant	N°pfmdr1-86_wild	OR (C.I 95 %)	P-value
**MSP1**	K1				
	Yes	7	247	1.12 (1.07-1.17)	0.993
	No	0	31	-	-
	MAD20				
	Yes	5	238	0.83 (0.52-1.35)	0.310
	No	2	40	-	-
	RO33				
	Yes	6	249	1.06 (0.94-1.94)	0.744
	No	1	29	-	-
**MSP2**	FC27				
	Yes	4	191	0.83 (0.43-1.58)	0.520
	No	3	87	-	-
	3D7				
	Yes	5	192	1.03 (0.64-1.66)	0.894
	No	2	86	-	-

**Figure 1:**
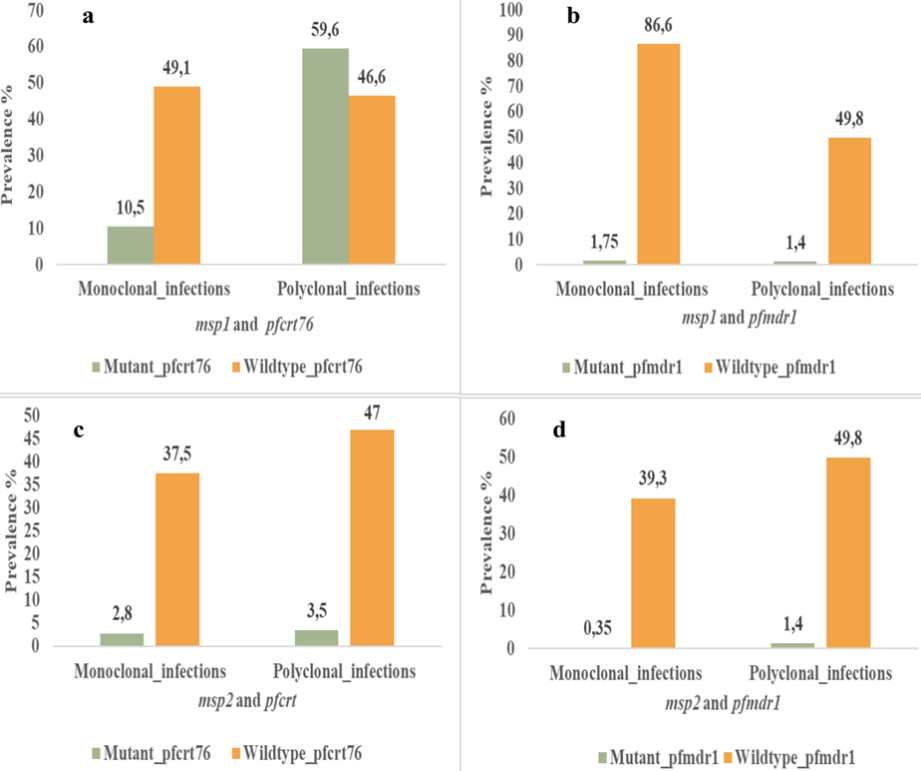
distribution of the prevalence of *msp1/msp2* monoclonal and polyclonal infections in association with the *pfcrt76*T and *pfmdr1*-86 resistance genes

**Clonality of *Plasmodium falciparum* infection and chloroquine resistance genes (*pfcrt76*T and *pfmdr1*-86Y):** the prevalence of monoclonal *msp1* infection was 49.1% for the wild-type *pfcrt76* gene and 10.5% for the mutant *pfcrt76* gene (Figure 1A). The prevalence of the mutant and wild-type *pfmdr1*-86 genes associated with monoclonal *msp1* infections was 1.75% and 86.6%, respectively (Figure 1B). Among polyclonal *msp1*-infected participants, 59.6% had a mutation in the *pfcrt76*T gene, and 1.4% had a mutation in the *pfmdr1*-86Y gene. No significant association was found between monoclonal *msp1* infections and mutations in the *pfcrt76*T (p=1) and *pfmdr1*-86Y (p=0.464) genes. Similarly, polyclonal-infected participants were unlikely to develop mutations in the *pfcrt76*T (p=0.300) and *pfmdr1*-86Y (p=1) genes. Regarding the *msp2* gene, only 2.8% of monoclonal *msp2* individuals harbored mutations in the *pfcrt76*T gene, while 37.5% harbored the wild-type *pfcrt76*T gene (Figure 1C). A solitary individual (0.35%) monoclonal to *msp2* exhibited a mutation in the *pfmdr1*-86Y gene (Figure 1D). There was no significant association between monoclonal or polyclonal *msp2* infection and the prevalence of the *pfcrt76*T (p=0.704) or *pfmdr1*-86Y (p=0.188) gene.

**Figure 2:**
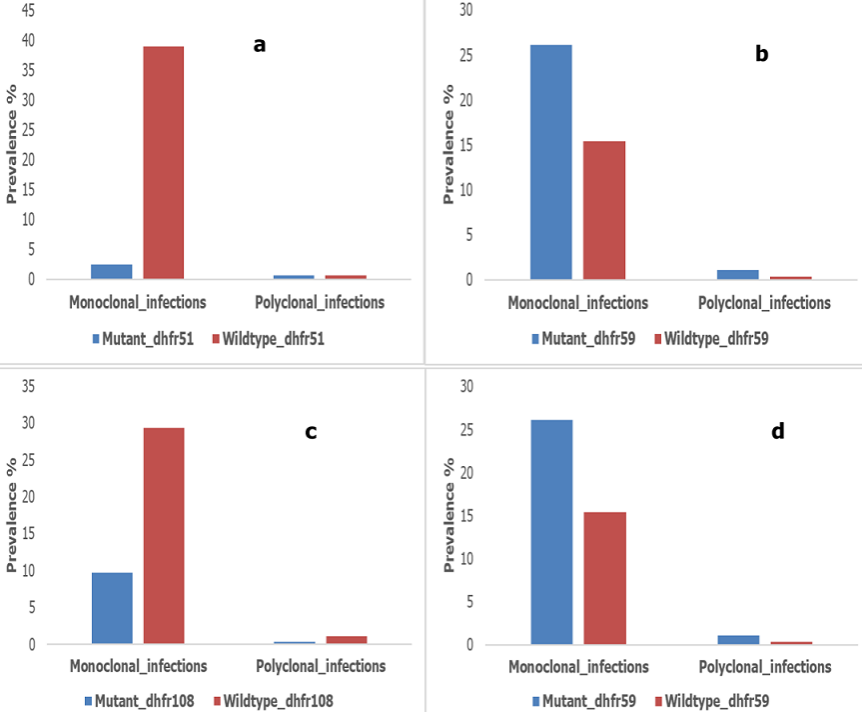
distribution of the prevalence of *msp1/msp2* monoclonal and polyclonal infections in association with the *dhfr*51, *dhfr*59, *dhfr*108 and 37 resistance genes

**Clonality of *Plasmodium falciparum* infection and sulfadoxine pyrimethamine resistance genes (*DHFR*/*DHPS*):** the prevalences of wild-types *dhfr* 51 [OR=0.35; CI (0.17-0.70); p<0.001] and *dhps*437 [OR=1.4; CI (1.05-1.87); p=0.031>] were significantly associated with monoclonal *Plasmodium falciparum* infection. Mutants and wild-type *dhfr*59 [OR=1.27; CI (0.94-1.70); p=0.105] and *dhfr* 108 [OR=0.91; CI (0.65-1.28); p=0.597] were not associated with monoclonal infection (Figure 2).

**Relationships between *msp1* and *msp2* alleles and SP resistance genes:** the prevalences of mutations at the *dhfr51, dhfr59, dhfr108* and *dhps*437 positions associated with K1, MAD20 and RO33 allele carriers were not statistically different (Table 3). Only the prevalences of mutant *dhfr*59 of K1[OR=0.97; CI (1.10-1.06); p=0.625] and RO33 [OR=1.02; CI (0.92-1.08); p=0.951] carriers were high but not significantly associated the development of resistance. Complexity of infection and antimalarial resistance genes: The complexity of infection (COI) in individuals with mutations in the *pfcrt76*T gene was 2.7 (95% CI 2.3-3.1) compared with 2.4 (95% CI 2.3-2.5) in those without mutations. This difference was statistically significant (p=0.035) (Figure 1A). For the mutant *pfmdr1*-86Y gene, the COI was 2.3 (95% CI 2.0-2.7) compared with 2.4 (95% CI 2.3-2.5) for the nonmutant *pfmdr1*-86 gene. Analysis of variance (ANOVA) revealed no significant difference between the *pfmdr1*-86 mutant gene and the wild type (Figure 1B). Figure 4 shows the mean COI in relation to mutants *dhfr*51 (Figure 4A), *dhfr*59 (Figure 4B), *dhfr*108 (Figure 4C) and *dhps*437 (Figure 4D) genes. The differences in mean complexity of infection (mCOI) between individuals with mutations in the *dhfr*51I (p=0.103), *dhfr*59R (p=0.073), *dhfr*108A (p=0.701) and *dhps*437G (p=0.444) genes and those with non-mutated genes were not significant.

**Table 3:** relationship between mutant/wild type *dhfr*51, *dhfr*59, *dhfr*108, *dhps*437 genes and *msp1/msp2* alleles distribution, analyzed by logistic regression

Polymorphic genes	Resistance genes	Prevalences of mutant genes	OR (C.I 95%)	P- value
**msp1**	**dhfr51**			
K1		14.33 % (n=40)	1.02 (0.92-1.13)	0.679
MAD20		12.2 % (n=34)	0.89 (0.75-1.05)	0.208
RO33		13.97 % (n=39)	0.98 (0.88-1.10)	0.844
**msp2**				
3D7		11.11 % (n=31)	1.02 (0.82-1.26)	0.835
FC27		11.11 % (n=31)	1.03 (0.83-1.27)	0.752
**msp1**	**dhfr59**			
K1		51.62 % (n=144)	0.97 (0.10-1.06)	0.626
MAD20		49.82 % (n=139)	1.0 (0.90-1.10)	0.994
RO33		52.32 % (n=146)	1.02 (0.92-1.08)	0.951
**msp2**				
3D7		41.93 % (n=117)	1.09 (0.93-1.28)	0.262
FC27		38.35 % (n=107)	0.91 (0.77-1.06)	0.244
**msp1**	**dhfr108**			
K1		22.93 % (n=64)	1.01 (0.12-1.11)	0.750
MAD20		49.82 % (n=139)	1.0 (0.90-1.10)	0.572
RO33		22.58 % (n=63)	0.98 (0.89-1.08)	0.814
**msp2**				
3D7		18.27 % (n=51)	1.05(0.88-1.25)	0.569
FC27		17.92 % (n=50)	1.03 (0.87-1.24)	0.676
**msp1**	**dhps437**			
K1		21.50 % (n=60)	1.04 (0.96-1.13)	0.351
MAD20		21.14 % (n=59)	1.08 (0.98-1.19)	0.160
RO33		20.78 % (n=58)	0.99 (0.9O-1.09)	0.942
**msp2**				
3D7		16.48 % (n=46)	1.03 (0.86-1.23)	0.744
FC7		15.41 % (n=43)	0.95 (0.78-1.16)	0.655

**Figure 3:**
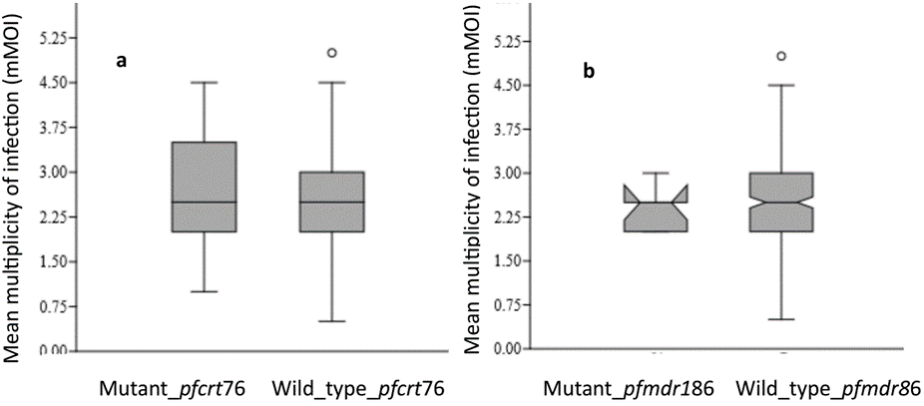
mean complexity of infection (mCOI) associated with mutant/wild type *pfcrt76* (a) and *pfmdr1*-86 (b)

**Figure 4:**
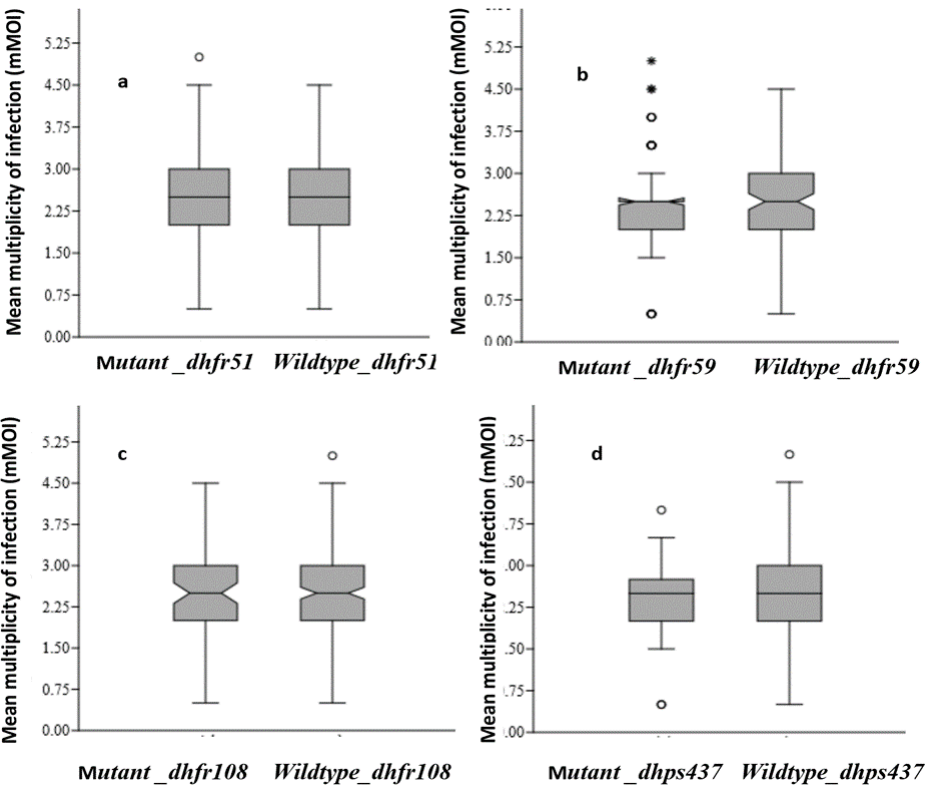
mean complexity of infection (mCOI) associated with mutant/wild-type *dhfr*51 (a), *dhfr*59 (b), *dhfr*108 (c) and *dhps*437 (d)

## Discussion

The characterization of different clones of malaria parasites and monitoring of the genes associated with antimalarial drug resistance are crucial for malaria control and elimination efforts. In this study, we investigated the relationships between genetic polymorphisms alleles, and genes associated with resistance to chloroquine and sulfadoxine-pyrimethamine.

The present study revealed a high multiple infection with *msp1* (K1, MAD20, RO33) and *msp2* (3D7, FC27) alleles. The majority of infections exhibited mixed alleles. The mean complexity of infection was estimated at 2.3 (95% CI 2.2-2.4). This observation confirmed the endemicity of the region where previous studies had already mentioned the high level of genetic polymorphism [37,38]. In our study, we found that the prevalences of the mutant genes *pfcrt76*T and *pfmdr1*-86Y, which confer resistance to chloroquine and amodiaquine were 4.2% and 7.3%, respectively. Theses prevalences were low compared to previous studies in Burkina Faso [33,39]. This result can be explained by the decreasing of the medicine pressure due to the discarding of chloroquine and the introduction of the combination-based artemisinin therapy [40]. The prevalences of the *dhfr*51I, *dhfr*59R, *dhfr*108A and *dhps*437G mutations, which are associated with resistance to sulfadoxine-pyrimethamine, were 15.4%, 57.2%, 24.9% and 22.8%, respectively. These prevalences were very low compared with those obtained by Geiger *et al*. [41] in the same locality. These results are indicative of an increase in sulfadoxine-pyrimethamine sensitivity, supported by numerous works including those by Ndiaye *et al*. 2013 [42] in Senegal and Tahita *et al*. 2015 [43] in Burkina Faso. We did not observe in this study any relationship between antimalarial resistance genes and the distribution of the *msp1* and *msp2* alleles. This is based on the fact that the prevalence of chloroquine and sulfadoxine-pyrimethamine resistance genes did not vary significantly according to the allelic forms of the *MSP1* and *MSP2* genes. This would mean that antimalarial drug resistance is not specifically carried by any family of *msp1* alleles (K1, RO33 or Mad20) or *msp2* alleles (FC27, 3D7). Similar observations regarding chloroquine resistance markers in the Nanoro area of Burkina have already been made by Sondo *et al*. who were only interested in determining the relationship between genetic polymorphism and chloroquine resistance genes, specifically *pfcrt76*T and *pfmdr1* [28]. This can be explained by the fact that the frequencies of the different *msp1*/*msp2* alleles obtained in this study were not significantly different, so that each allele was likely to be affected by a mutation conferring resistance to either chloroquine or sulfadoxine-pyrimethamine. Our results are also supported by the absence of significant association between *MSP1/MSP2* monoclonal or polyclonal infection and chloroquine or sulfadoxine-pyrimethamine resistance markers. Previous studies have shown that the distribution of mutants *pfcrt76, pfmdr1, dhfr* and *dhps* alleles is independently linked with the number of *msp1* or *msp2* parasite clones taken individually [9,44]. In other words, mutations conferring chloroquine and sulfadoxine-pyrimethamine resistance are not associated with the number of *msp1* or *msp2* genotypes although it was expected that monoclonal variants, which are monomorphic, would be more susceptible to mutations such as those associated with antimalarial drug resistance [45]. In our opinion, the low prevalence of monoclonal infections obtained in this study may be the reason for this non-association. In the case of chloroquine resistance, Tinto *et al*. found that the discontinuation of this drug in most endemic countries did not affect the distribution of polymorphic gene alleles in these areas, either before or after the discontinuation [22]. The intrinsic and extrinsic phenomena leading to the emergence and spread of resistance can occur independently of the *msp1* and *msp2* strains. Other factors such as, the use of substandard drugs, uncontrolled drug prescribing and demographic pressure, also contribute to the development and spread of resistance [39]. In this study, the mean complexity of infection which represents the average number of *msp1* and *msp2* genotypes, was higher in individuals with a mutation in the *pfcrt76*T gene compared to those who did not have a mutation in this gene. This observation suggests that the pfcrt gene may be sensitive to the intensity of malaria transmission, a result consistent with the observation of Talisuna *et al*. [9] in Uganda, who had observed a high frequency of mutant *pfcrt76*T alleles associated with a high number of parasite clones in high transmission area. For the mutation in the *pfmdr1* gene, no association was found with the mean complexity of malaria infection. We believe that the specificity of the pfcrt gene, which is strongly associated with resistance to chloroquine compared to the *pfmdr1* gene, may explain this difference [46]. Regarding the genes involved in sulfadoxine-pyrimethamine resistance, we did not observe any correlation with the average number of genotypes. The weak association between genetic diversity and antimalarial resistance markers is also supported by the fact that the study was conducted in a holoendemic area where malaria transmission is high, while it is reported that the emergence and spread of antimalarial drug-resistant molecules has always occurred in areas of low malaria transmission, such as Southeast Asia and South America [7]. In fact, in these areas, fewer clones of *Plasmodium falciparum* are circulating, and the slightest presence of the parasite can lead to severe clinical cases [9]. Treatment of these low-density parasites exerts some drug pressure, leading to the emergence of resistance [26]. The genetic variability of *Plasmodium falciparum*, which depends on the level of transmission intensity, does not directly influence antimalarial resistance, but rather clinical and epidemiological factors such as the multiplicity of infection and the immunity of the human population [19].

Our study has many limitations. We only used the complexity of infection for the assessment of malaria transmission intensity, although its estimation may also include other determinants such as entomological inoculation rate and malaria prevalence. Clinical aspects and the use of substandard drugs, which could undoubtedly influence the development of antimalarial drug resistance, were not investigated in this study.

## Conclusion

Our study has shown that the genetic diversity of *Plasmodium falciparum* does not impact the mutations associated with chloroquine and sulfadoxine-pyrimethamine resistance. However, the complexity of infection was high, with individuals carrying the mutant *pfcrt76*T gene. Although these findings could be valuable for informing malaria control strategies, further research, particularly on clinical/epidemiological aspects, is needed to provide additional insights into the evolution of malaria drug resistance.

### What is known about this topic

- *Among the Plasmodium falciparum polymorphism genes, msp1 and msp2 are the most studied for their ability to characterize genetic diversity;*
- *Mutant strains of pfcrt76 and pfmdr1-86 are associated with Plasmodium falciparum mono infection;*
- *The dhfr (51,59,108) and dhps (437,540) gene mutations are associated with sulfadoxine-pyrimethamine resistance*.

### What this study adds

- *No relationship was found between msp1/msp2 alleles distribution and chloroquine (pfcrt76, pfmdr1-86) or sulfadoxine-pyrimethamine (dhfr51, dhfr59, dhfr108, dhps437) resistance genes;*
- *Plasmodium falciparum mono and poly infections were associated with chloroquine (pfcrt76, pfmdr1-86) or sulfadoxine-pyrimethamine (dhfr51, dhfr59, dhfr108, dhps437) resistance genes;*
- *The complexity of infection is significantly correlated with the prevalence of the mutant pfcrt76T gene*.
